# Prosthetic heart valves in pregnancy: a systematic review and meta-analysis protocol

**DOI:** 10.1186/2046-4053-3-8

**Published:** 2014-01-21

**Authors:** Claire M Lawley, Samantha J Lain, Charles S Algert, Jane B Ford, Gemma A Figtree, Christine L Roberts

**Affiliations:** 1 Clinical Population Perinatal Health Research Group, The Kolling Institute, University of Sydney at Royal North Shore Hospital, Herbert Street, St Leonards, New South Wales 2065, Australia; 2 Department of Cardiology, Royal North Shore Hospital, Herbert Street, St Leonards, New South Wales 2065, Australia

## Abstract

**Background:**

Advances in surgical technique, prosthetic heart valve design, and anticoagulation have contributed to an overall improvement in morbidity and mortality in women with heart valve prostheses as well as increased feasibility of pregnancy. Previous work investigating the pregnancies of women with prosthetic valves has been directed largely toward understanding the influence of anticoagulation regimen. There has been little investigation on maternal and infant outcomes. The objective of this systematic review will be to assess the outcomes of pregnancy in women with heart valve prostheses in contemporary populations.

**Methods/Design:**

A systematic search of Medline, Embase, Cumulative Index to Nursing and Allied Health Literature (CINAHL), and the Cochrane Library will be undertaken. Article titles and abstracts will be evaluated by two reviewers for potential relevance. Studies that include pregnancies occurring from 1995 onwards and where there are six or more pregnancies in women with heart valve prostheses included in the study population will be reviewed for potential inclusion. Primary outcomes of interest will be mortality (maternal and perinatal). Secondary outcomes will include other pregnancy outcomes. No language restrictions will be applied. Methodological quality and heterogeneity of studies will be assessed. Data extraction from identified articles will be undertaken by two independent reviewers using a uniform template. Meta-analyses will be performed to ascertain risk of adverse events and, where numbers are sufficient, by type of prosthesis and location as well as other subgroup analyses.

**Discussion:**

Estimates of the risk of adverse events in recent pregnancies of women with heart valve prosthesis will provide better information for counselling and decision making. Given the improvements in prognosis of heart valve prosthesis recipients and the paucity of definitive data regarding optimal pregnancy management for these women, review of this topic is pertinent.

**Review registration:**

This protocol has been registered with the international prospective register of systematic reviews (PROSPERO) as number CRD42013006187, accessible online at http://www.crd.york.ac.uk/PROSPERO/display_record.asp?ID=CRD42013006187#.Utk7qNJ9Lf8.

## Background

The etiology of valvular heart disease and management of congenital heart disease in young women continues to change. Advances in surgical technique, prosthetic heart valve design, and anticoagulation have contributed to an overall improvement in morbidity and mortality [1-4]. The number of women with heart valve prostheses counselled explicitly against pregnancy is decreasing with improvement in understanding of what conditions and cardiac parameters constitute high risk of adverse events during pregnancy [5,6]. The focus is shifting to provision of informed decision making around the risk pregnancy might place on women and their babies. With these changes in mind, understanding the outcomes of pregnancies in women who have heart valve prostheses in the contemporary setting is of increasing relevance.

During normal pregnancy, there is an increase in hemodynamic load, which continues to rise during labor. This is as a result of increases in stroke volume and heart rate, increasing cardiac output by an estimated 30% to 40%, combined with a decrease in total peripheral resistance, leading to a decrease in blood pressure [7-9]. Pregnancy is a pro-coagulant state due to an elevation in circulating pro-coagulant factors and maternal hormones, leading to decreases in prothrombin time, activated partial thromboplastin time, thrombin time, and international normalized ratio (INR) [10,11]. Tolerance of these hemodynamic and coagulatory changes in women with pre-existing heart disease, including those with bioprosthetic and mechanical heart valve prostheses, is known to vary with underlying cardiac function and etiology of cardiac disease [6].

Previous work surrounding heart valve prostheses in pregnancy has been largely directed toward understanding the influence of anticoagulation type in the setting of mechanical heart valve prostheses [12-16]. A systematic review published in 2000, including 976 women who had 1,234 pregnancies, focused on maternal and fetal complications associated with various anticoagulation regimens [17]. The review included studies with pregnancies occurring from 1966 to 1997. As such, a large number of study participants (433/976) had older-generation and more thrombogenic cage-and-ball heart valve prostheses. Pooled analysis from this work demonstrated higher rates of fetal malformation in those women treated with oral anticoagulation in the first trimester (6.4%, confidence interval (CI) 95% 4.6%-8.9%, of pregnancies) as compared with where heparin was used in the first trimester (3.4%, CI 95% 1.4%-7.7%, of pregnancies) [17]. A higher risk of thromboembolic complications was noted with heparin use. Another more recent review (2011) using pooled data from 959 pregnancies receiving oral anticoagulation throughout pregnancy and 285 pregnancies receiving unfractionated heparin in the first trimester found an incidence of maternal thromboembolic complications in 3.9% and 9.5% of pregnancies in each group, respectively [15].

In the setting of mechanical heart valve prostheses in pregnancy, there is consensus in current international guidelines that one of three anticoagulation regimens may be used following assessment of maternal risk factors and preference: oral anticoagulation throughout pregnancy, oral anticoagulation with replacement by low-molecular-weight heparin or unfractionated heparin during weeks 6 to 12, or low-molecular-weight heparin or unfractionated heparin throughout pregnancy. Each of these regimens requires counseling around risk and judicious monitoring throughout, including INR and anti-factor Xa levels where applicable [5,18,19]. The Royal College of Obstetricians recommends that women be offered a choice of one of these three regimens with education around the risks and benefits of each [20].

Bioprosthetic heart valves avoid the need for anticoagulation during pregnancy [19,21]. However, their use in younger patients has previously been limited due to the increased need for re-replacement compared with their mechanical counterparts [22,23]. This has been demonstrated in young women specifically, with 82% (CI 95% 62%-92%) of women with bioprosthetic valve prostheses requiring replacement at 10 years as opposed to only 29% (CI 95% 17%-39%) of women with mechanical valves [22]. Despite initial suggestions, recent work has not shown an increase in the rate of bioprosthetic valve deterioration in women undertaking pregnancies as opposed to women who do not [24,25]. With decreasing mortality and morbidity associated with valve re-replacement, international guidelines suggest that bioprosthetic valves be considered when heart valve replacement is required in women who may wish to become pregnant [5,26].

Little work has been done exploring the population of contemporary heart valve recipients undertaking pregnancy. Specifically, other than anticoagulant type [12-17], there has been little investigation around rates and risk factors for maternal and infant adverse events. Given the changes in heart valves used, improvement in prognosis of contemporary heart valve prosthesis recipients, and the paucity of data in regarding the outcomes of pregnancies in these women, review of the studies in this area is warranted.

### Objectives

Primary: To assess the risks of adverse outcomes of pregnancy among women with a prosthetic heart valve(s) in the contemporary setting.

Secondary: To assess the risks and relative risks of adverse outcomes of pregnancy in women with a prosthetic heart valve(s) by prosthesis type or location or both.

## Methods/Design

### Study registration

This protocol has been registered with the international prospective register of systematic reviews (PROSPERO) as number CRD42013006187.

The systematic review protocol has been conducted and reported by using the Preferred Reporting Items for Systematic Reviews and Meta-Analyses (PRISMA) [27] where applicable and the Meta-analysis of Observational Studies in Epidemiology (MOOSE) reporting guidelines [28].

### Outcomes of interest

Primary outcomes:

1) Maternal mortality

2) Any pregnancy loss

a. Any loss of pregnancy, including miscarriage/stillbirth/termination of pregnancy [29] (or as defined by study)

3) Perinatal mortality [29]

a. Stillbirth: Fetal death *in utero* at least 22 weeks of gestation [29] (or as defined by the study)

b. Neonatal mortality: Death in the first 28 days of extra-uterine life [29]

c. Perinatal mortality: Stillbirth or neonatal mortality

Secondary outcomes:

1) Adverse maternal outcomes

a. Any thromboembolic events, including

i. Stroke/Transient ischemic event

ii. Valve thrombosis

iii. Other

b Any obstetric hemorrhage, including

i. Antenatal hemorrhage

ii. Postpartum hemorrhage

c. Cardiovascular compromise (as defined by study)

d. Valve deterioration (bioprosthetic valves only, as defined by study)

e. New arrhythmia

f. Infective endocarditis

g. Myocardial infarction

h. Pregnancy hypertension, including gestational hypertension, pre-eclampsia, and eclampsia

2) Labor and delivery outcomes (including those subject to clinical decision making)

a. Mode of delivery

3) Adverse birth outcomes

a. Preterm birth

Delivery before 37 weeks of gestation

b. Small for gestational age

Less than tenth birth weight percentile for sex and gestational age

c. Low birth weight

Birth weight less than 2,500 grams

d. Infant admission to neonatal intensive care unit

e. Congenital malformation

### Search strategy for identification of studies and methods of review

A systematic search of Medline, Embase, Cumulative Index to Nursing and Allied Health Literature (CINAHL), and the Cochrane Library will be undertaken to identify relevant studies published between 1995 and May 2013. Search terms will include “pregnancy” AND (“heart valves” OR “heart valve replacement” OR “heart valve prosthesis” OR “heart valve prosthesis implantation”). The “explode” function will be used in each case. Searches will be limited to studies of humans and peer-reviewed articles. Language restrictions will not be applied, and every effort will be made to obtain translations; articles unable to be translated will be reported. Duplicates will be removed.

### Eligibility criteria for consideration of inclusion

Study types:

Studies that report outcomes of women with prosthetic valves undertaking pregnancy:

•Randomized controlled trials

•Clinical trials

•Cohort studies

•Cross-sectional studies

•Unselected case series

Studies that compare outcomes for women with prosthetic valves by valve location or type:

•Randomized controlled trials

•Clinical trials

•Cohort studies

•Case–control studies

Control or comparison groups are not necessary to the primary objective of estimating risk among women with valve prosthesis. It is anticipated that most of the studies identified for consideration will be case series.

Populations: Populations of pregnant women that include women with prosthetic heart valves.

Comparators:

Where studies differentiate between mechanical and biological valve prosthesis, or between valve location (that is, mitral, aortic, pulmonary, and tricuspid), applicable to the secondary objective; relative risks by prosthesis type or location or both, relative risks of adverse events will be calculated by using:

•Biological prosthesis as the denominator for calculating relative risk compared with mechanical

•Presence of mitral valve prosthesis as the denominator for calculating relative risk compared with other valve locations

Study criteria:

•Include pregnancies occurring from 1995 onwards only.

•Contain at least six pregnancies in women with heart valve prostheses in the study population. This was chosen as it has been used in a systematic review exploring anticoagulation regimens during pregnancy in women with heart valve prostheses [17].

•Study population should have fewer than 5% of women with a Starr-Edwards (cage-and-ball) heart valve prosthesis. This was a pragmatic decision. Owing to high thrombogenic complication rates, cage-and-ball prostheses are no longer implanted [30,31] and therefore are not relevant when evaluating in the contemporary setting. Consequently, where the study population consists of not more than 20 women, if one (5%) or more participants has a cage-and-ball valve, the study will be excluded.

•Where a case series is presented, participants have not been selected due to the occurrence of an adverse event (for example, valve thrombosis during pregnancy).

•Not a conference abstract and unpublished study.

Exposure of interest: Pregnancy in women with a heart valve prosthesis.

### Screening of studies

Article titles and abstracts will be evaluated by two reviewers for potential relevance. Where there is disagreement at this stage, the article will remain included until the full text is reviewed prior to a decision. In this process, articles relating to other aspects of valvuar heart disease and pregnancy but not specific to maternal vavular prostheses will be excluded (for example, articles related to fetal cardiac disease or basic science). Articles identified through reference lists of included studies and relevant systematic reviews will be considered for inclusion on the basis of their title.

At least two independent reviewers will assess all articles identified in the screening process for potential inclusion, including assessment of methodological quality as outlined below. Where information pertinent to inclusion criteria is not contained within the article text, the effort will be made to contact the listed corresponding author. Where no reply is received, the article will be excluded. Consensus between the two authors undertaking review of the study will need to be reached before the article is included. In the event that a consensus is not reached, a third reviewer will be involved as an arbitrator. A flow chart of the study selection procedure will be prepared and a log of rejected studies maintained.

### Data extraction

Data extraction from identified articles will be undertaken by two independent reviewers using a uniform template. Discrepancies will be resolved by discussion and, where applicable, arbitration by a third reviewer.

The following information will be extracted:

Study characteristics: authors, year of publication, study design, location, and time period of included pregnancies

Population characteristics: number of participants, number of pregnancies, maternal age, and parity

Heart valve characteristics: number of mechanical valves, number of bioprosthetic valves, implanted valve type, implanted valve location, and anticoagulation regimen

Adverse outcomes: frequency of adverse outcomes as outlined above.

### Assessment of methodological quality

It is thought likely that the only randomized studies eligible for inclusion will be randomized control trials assessing different valve types. The risk of bias in randomized studies will be assessed by using the Cochrane Collaboration’s tool for assessing risk of bias [32]. This tool provides a model to evaluate the risk of bias across a number of domains: how a study selects participants, measures performance, blinds participants and investigators, explores attrition, and reports findings. Each domain for each study will be allocated a ranking of “low”, “unclear”, or “high” risk of bias, in accordance with the Cochrane Collaboration’s approach, by two separate reviewers. Where there is a discrepancy between the two reviewers, a third reviewer will be used as an arbitrator.

Included non-randomized studies may or may not have a comparison group. To assess the risk of bias within included these studies, the methodological quality of potential studies will be assessed by using the Newcastle-Ottawa scale (NOS) for assessing the quality of non-randomized studies in meta-analyses [33]. The NOS for case–control and cohort studies will be adapted (Table 1) to meet the specific needs of this systematic review. The cohort scale will be modified for use in case series [33]. Using the NOS, studies will be awarded a maximum of nine points on items related to the selection of the study groups, the comparability of the groups, and the ascertainment of outcome of interest. Using this modified score, case series will be eligible for a maximum of six points. This will be undertaken by two separate reviewers. Where there is disagreement, a third reviewer will be used as an arbitrator.

**Table 1:** **Adapted Newcastle-Ottawa scale**[33]**for “Prosthetic heart valves in pregnancy: a systematic review and meta-analysis protocol”**

**Criteria**	**Star allocated (maximum 9 stars)**^**a**^
Selection	
1) Representativeness of the exposed cohort	
a) Population truly representative of pregnant women with prosthetic heart valves	★
b) Somewhat representative of the population of pregnant women with prosthetic heart valves	★
c) Selected group of users (for example, referral hospital patients)	-
d) No description of the derivation of the cohort	-
2) Selection of the non-exposed cohort^b^	
a) Drawn from the same community as the exposed cohort	★
b) Drawn from a different source	-
c) No description of the derivation of the non-exposed cohort	-
d) Not applicable	-
3) Ascertainment of exposure	
a) Secure record (for example, medical records)	★
b) Structured interview	★
c) Written self-report	-
d) No description	-
4) Demonstration that outcome of interest was not present at start of study^c^	
a) Yes	★
b) No	-
c) Not applicable	-
Comparability	
Comparability of cohorts on the basis of the design or analysis	
a) Study controls for maternal age (select the most important factor)	★
b) Study controls for any additional factor (type of valve, valve location, anticoagulation regimen)	★
c) Not applicable	-
Outcome	
1) Assessment of outcome	
a) Independent blind assessment	★
b) Record linkage	★
c) Self-report	-
d) No description	-
2) Was follow-up long enough for outcomes (as defined by study) to occur?	
a) Yes	★
b) No	-
3) Adequacy of follow up of cohorts	
a) Complete follow up (all subjects accounted for and no missing data)	★
b) Subjects lost to follow-up unlikely to introduce bias - small number lost - ≥ 80%	★
c) Follow-up rate < 80%	-
d) No statement	-

^a^A study can be awarded a maximum of one star for each numbered item within the selection and outcome categories. A maximum of two stars can be given for comparability. ^b^For example, women without a heart valve prosthesis undertaking pregnancy. Likely to be “not applicable” for some study types, including case series. ^c^Suggested primary outcomes: maternal mortality, any pregnancy loss, and perinatal mortality.

### Data analysis and presentation

A table with descriptive information for each study will be produced Additional file 1: Table S1. From extracted data, the risk of outcomes for the primary objective will be calculated by dividing the total number of outcome occurrences by the total number of pregnancies or births to women with a heart valve prosthesis. The risk of maternal mortality and any pregnancy loss will be expressed as the proportion of the total number of pregnancies (including miscarriages, terminations, stillbirths, and live births). The risk of perinatal death and secondary adverse birth outcomes (as opposed to pregnancy outcomes) will be expressed as a proportion of the pregnancies beyond 22 weeks of gestation or 500 grams or resulting in a live birth [29]. It is anticipated that these denominators may not always be clearly articulated, potentially constraining the process to what is reported in each study.

Subgroup analysis of primary and secondary outcome relative risks by valve location and valve type will be undertaken if reported by at least two studies, each with at least six pregnancies in the subgroup. Comprehensive Meta-Analysis (version 2.0) software (Biostat, Englewood, NJ, USA) be used for the data analysis. This software enables pooling of risks as well as of relative risks, making it suitable for our primary objective, especially as it is anticipated that the majority of studies will be case series. Pooled risks will be calculated by using a random effects model, with variance calculated by using a logit conversion. Graphic summaries of individual study estimates and overall estimates will be produced. Statistical uncertainty will be assessed by using 95% confidence intervals around risk estimates.

Where applicable, heterogeneity of effect for studies within a meta-analysis will be assessed with the I^2^ statistic. Study heterogeneity will be explored by categorization of the study design, the year of publication, the time period within which pregnancies occur, and population characteristics (ethnicity, age range, etiology of underlying disease, type and location of heart valve prosthesis, and anticoagulant regimen). It is expected that study characteristics will vary and that random effects models will be appropriate for estimating overall event risks.

In general, the strength of evidence will be assessed with respect to the study designs, the methodological quality of the individual studies, the consistency of the results across studies, and, for studies with a comparison or control group, the strength of associations. More specifically, given the likelihood that most studies will be uncontrolled case series, the strength of evidence will be assessed primarily by the width of the confidence interval around pooled outcome rates. Consistency of effect will also be important both as demonstrated visually in the plots and as quantified by the I^2^ statistic.

## Discussion

Improved care for chronic diseases and delayed age of childbearing has contributed to an increase in the number of pregnant women with concurrent medical conditions, including valvular heart disease. The proposed systematic review is of importance in the context of global pressure to improve maternal and infant health, including the evaluation of pregnancies in subgroups of women with co-morbidities. In Australia, this is seen through the prioritization of research work encompassing “Healthy start to life for all Australians” [20].

Meta-analyses of observational studies present challenges because of inherent biases within different study designs [34]. Nevertheless, they help understanding and quantify variation in results between studies [28]. In the context of predominantly observational studies, it is thus essential that a rigorous protocol be designed to address the outcome of pregnancies in women with heart valve prostheses.

Through exploration of the outcomes of pregnancies in women with heart valve prosthesis in the contemporary setting (1995 onwards), this systematic review will provide estimates of the risk of adverse events in these pregnancies. It is hoped that this information will improve the understanding of risk factors for poor maternal, pregnancy, and infant outcomes, thereby providing information for clinical decision making and patient counselling. It is timely that this work is undertaken given the developments in heart valve prosthesis technology, overall improvement in prognosis of young women with a heart valve prosthesis, and increases in the number of women with congenital heart disease reaching reproductive age.

## Abbreviations

CI: confidence interval; INR: international normalized ratio; NOS: Newcastle-Ottawa scale.

## Competing interests

The authors declare that they have no competing interests.

## Authors’ contributions

CR and GF helped to conceive of the study and were involved in the design of the above review protocol and in drafting and revising the manuscript. CL, SL, CA, and JF were involved in the design of the above review protocol and in drafting and revising the manuscript. All authors read and approved the final manuscript.

## Supplementary Material

Additional file 1: Table S1 Sample of table to record descriptive information extracted from each included study. Click here for file
