# Supplementary material for: Prosthetic heart valves in pregnancy: a systematic review and meta-analysis protocol
Source: Syst Rev. 2014 Jan 21;3:8. doi: 10.1186/2046-4053-3-8 (PMC3913632; doi:10.1186/2046-4053-3-8)
Supplement: Additional file 1: Table S1 — Sample of table to record descriptive information extracted from each included study. [file 2046-4053-3-8-S1.docx]

**Additional file 1: Table S1.** Sample of table to record descriptive information extracted from each included study

| Study | Subgroup | Number of women valve prostheses | Number of pregnancies | Number pregnancies >22 weeks | Maternal mortality (n) | Any pregnancy loss (n) | Perinatal mortality (n) | Miscarriage (n) | Termination of pregnancy (n) | Stillbirth (n) | Neonatal death (n) | Live birth (n) | Preterm birth (n) | Small for gestational age (n) | Low birth weight (n) | Infant admission to NICU (n) | Congenital malformation (n) | Any thromboembolic event (n) | Ischemic stroke/TIA (n) | Valve thrombosis (n) | Other (n) | Any obstetric hemorrhage (n) | Antenatal hemorrhage (n) | Postpartum hemorrhage (n) | Cardiovascular compromise (n) | Valve deterioration (n) | New arrhythmia (n) | Infective endocarditis (n) | Myocardial infarction (n) | Pregnancy hypertension (n) | Spontaneous vaginal delivery (n) | Instrumental vaginal delivery (n) | Pre-labor caesarean section (n) | Intrapartum caesarean section (n) |
| --- | --- | --- | --- | --- | --- | --- | --- | --- | --- | --- | --- | --- | --- | --- | --- | --- | --- | --- | --- | --- | --- | --- | --- | --- | --- | --- | --- | --- | --- | --- | --- | --- | --- | --- |
| “A” | All* |  |  |  |  |  |  |  |  |  |  |  |  |  |  |  |  |  |  |  |  |  |  |  |  |  |  |  |  |  |  |  |  |  |
|  | Study A, subgroup 1† |  |  |  |  |  |  |  |  |  |  |  |  |  |  |  |  |  |  |  |  |  |  |  |  |  |  |  |  |  |  |  |  |  |
|  | Study A subgroup 2 ‡ |  |  |  |  |  |  |  |  |  |  |  |  |  |  |  |  |  |  |  |  |  |  |  |  |  |  |  |  |  |  |  |  |  |
| … |  |  |  |  |  |  |  |  |  |  |  |  |  |  |  |  |  |  |  |  |  |  |  |  |  |  |  |  |  |  |  |  |  |  |

* All pregnancies to women included in the study.

† e.g. pregnancies to women with a mechanical prosthesis in study A.

‡ e.g. pregnancies to women with a bioprosthesis in study A.
